# Alteration of interoceptive sensitivity: expanding the spectrum of behavioural disorders in amyotrophic lateral sclerosis

**DOI:** 10.1007/s10072-022-06231-4

**Published:** 2022-06-25

**Authors:** Pasquale Moretta, Myriam Spisto, Francesco Pio Ausiello, Rosa Iodice, Natascia De Lucia, Gabriella Santangelo, Luigi Trojano, Elena Salvatore, Raffaele Dubbioso

**Affiliations:** 1 Istituti Clinici Scientifici Maugeri IRCCS, Neurological Rehabilitation Unit of Telese Terme Institute, 82037 Telese Terme, Benevento, Italy; 2 Department of Neurosciences, Reproductive Sciences and Odontostomatology, University of Naples Federico II, Via Sergio Pansini, 5, 80131 Naples, Italy; 3 Department of Psychology, University of Campania Luigi Vanvitelli, Naples, Italy

**Keywords:** Interoception, Emotion, Behavioural, Cognition, HRV, Rehabilitation

## Abstract

**Background:**

Amyotrophic lateral sclerosis (ALS) is a neurodegenerative disorder with progressive loss of upper and lower motor neurons. Non-motor-symptoms, such as cognitive, emotional, autonomic, and somatosensory alterations, have been also described.

Interoception represents the link between the body and brain, since it refers to the ability to consciously perceive the physical condition of the inner body, including one’s heartbeat (i.e., interoceptive sensitivity, IS).

**Objectives:**

To evaluate IS in ALS patients by means of a well-established task: the heartbeat perception task. Moreover, we evaluated possible correlations between IS and neuropsychological, affective, and disease-related characteristics.

**Methods:**

Fifty-five ALS patients (mean-age = 60.3 ± 12.5 years; mean disease-duration = 20.9 ± 18.8 months) and 41 caregivers (CG) underwent the heartbeat perception task and an extensive evaluation of motor, cognitive, body awareness, affective, and emotion domains.

**Results:**

ALS patients showed lower IS than CG (0.68 ± 0.24 vs 0.82 ± 0.16; *p* = 0.003). Significant correlations were found between IS and self-reported measures of alexithymia (subscale of Toronto Alexithymia scale-20 “difficulties in describing feelings”; rho =  − .391, *p* = .003) and interoceptive awareness (subscale of Multidimensional assessment of interoceptive awareness “not worrying about pain”; rho = .405, *p* = .002). No significant differences were found on questionnaires for depression and anxiety between patients with ALS and their caregivers (*p* > .05).

**Conclusions:**

ALS patients show reduced interoceptive sensitivity that is associated with poorer ability to describe feelings and with lower focalization on pain, regardless of cognitive and motor impairment. Alteration of interoception may represent a specific behavioural sign within the spectrum of emotion processing deficits described in ALS patients.

**Supplementary Information:**

The online version contains supplementary material available at 10.1007/s10072-022-06231-4.

## Introduction

Amyotrophic lateral sclerosis (ALS) is a neurodegenerative disease mainly affecting upper and lower motor neurons, with consequent motor impairment. In the last two decades, non-motor symptoms, in the cognitive, behavioural, and emotional domains, have been identified as well [1–4].

Cognitive impairment is reported in approximately 30–50% of patients with ALS [5] and may negatively impact on survival [6, 7] and caregiver burden [8]. Among patients with cognitive impairment, a relatively small proportion (about 10%) presents frontotemporal dementia, mostly the behavioural variant [5]. Cognitive deficits have been shown in different domains. Most consistently, executive dysfunction and verbal fluency deficits are reported, but recently impairments in language and memory have been identified in patients with ALS [4, 9]. Moreover, facial emotion recognition, judgments of emotional valence, and social cognition skills such as decision-making and theory of mind (ToM) may also be impaired [10]. About 30% of patients show behavioural alterations, mainly characterized by apathy and irritability [11]. The prevalence of depression in ALS varies considerably ranging from 5 to 30%, whilst anxiety occurs in 20–35% of patients [12].

Structural and functional neuroimaging studies showed significant cortical thinning and reduced functional activation in the pre-central gyrus and supplementary motor area [13–15] as well as in extra-motor regions such as the prefrontal and temporal cortices [15, 16]. Interestingly, the involvement of limbic areas, including the insula, has been recently described in the neurodegenerative process of the ALS [10, 17, 18].

These areas, and the insula in particular, are thought to be key regions for interoception [19–21] that is the perception of sensations from inside the body, related to the function of internal organs [22]. Moreover, it has been proposed that subjective “feeling states” are dependent on the process of interoception: the representation and contextualisation of somatic and visceral responses elicited by emotional stimuli. In ALS, many components of the emotional processing are altered [23], and the insula has been suggested as a key anatomical region involved in several non-motor symptoms, including depression, anxiety, apathy, anhedonia, and fatigue [24]. However, besides interoceptive brain centres, viscerosensory afferent pathways, mainly from the vagus nerve, play an important role in modulating interoception [25, 26]

Accuracy in interoception is expressed as interoceptive sensitivity (IS). IS can be measured by using the heartbeat detection task, that is the ability to detect sensations from one’s heart.

Impairment of the heartbeat detection task, and therefore of IS, has been found in neurological disorders characterized by altered emotion processing, such as Parkinson disease [27], vascular focal brain lesions [28], and multiple sclerosis [29]. To the best of our knowledge, no study has systematically evaluated IS in patients with ALS and its correlation with cognitive and behavioural aspect of disease.

On these bases, the present study had three main aims: (1) to assess interoceptive sensitivity in patients affected by ALS, by comparing scores obtained on the heartbeat perception task in patients and in their caregivers; (2) to ascertain possible relations of IS with behavioural and cognitive measures in patients with ALS; (3) to explore whether IS difficulties are related with severity of clinical disability and/or the rate of disease progression. We enrolled patients’ caregivers as the control sample to rule out possible confounding factors related to interoception assessment, such as depression and anxiety [30], since similar levels of psychological distress have been described in both groups [31, 32].

## Materials and methods

### Participants

This study included patients with ALS consecutively admitted to the ALS centre of the University Hospital Federico II of Naples in the period June 2020 to June 2021. Patients aged 18 years or more and met the “probable,” “probable laboratory-supported,” or “definite” diagnostic categories as per the revised El Escorial criteria for ALS [33]. Patients with history of neurologic disorders affecting cognition (major stroke, severe head injuries, mental retardation), alcohol dependence or drug dependence, severe mental illness, or use of high-dose psychoactive medications were not included in data analysis. In addition, patients with a diagnosis of frontotemporal dementia (FTD) [44], unable to communicate adequately, either verbally or by writing, were excluded.

Functional assessment of patients was performed by the Revised Amyotrophic Lateral Sclerosis Functional Rating Scale (ALSFRS-R). Disease onset (i.e., spinal vs bulbar onset) as well as disease phenotype, according to Chiò classification [34], were also recorded.

Moreover, we evaluated respiratory function, through spirometry performed with the patient sitting upright. Results for forced vital capacity (FVC) were expressed as a percentage of predicted value, from an average of three trials [35].

Genetic analysis was performed in all patients, exploring *C9orf72* repeat expansion and mutations of *SOD1*, *TARDBP*, and *FUS* genes. Routine magnetic resonance imaging (MRI) with a 3-T scanner was obtained for all patients.

All patients completed the cognitive, and psychological assessment specified below, the heartbeat counting task, and an assessment of interoceptive awareness.

We also recruited patients’ caregivers as control’s group. Only patient’s main informal caregivers were enrolled; professional caregivers were not included in the study. We excluded caregivers affected by any neurological, psychiatric, or other relevant clinical condition.

### Neuropsychological assessment and cognitive classification

To assess cognitive and behavioural profile, neuropsychologists with specific expertise in ALS assessment (M.S., F.P.A.) administered a multi-domain battery to all participants. For assessing global cognitive functioning both ALS patients and caregivers underwent the Italian versions of the Mini-Mental State Examination (MMSE) [36] and of the Edinburgh Cognitive and Behavioural ALS Screen (ECAS) [37], a rapid screening test (15–20 min), including an ALS-specific section (assessing executive functions, social cognition, verbal fluency and language; 0–100 points), and a non-ALS-specific section (that assesses memory and visuospatial abilities; 0–36 points). ECAS total score ranges from 0 (worst performance) to 136 (best performance). Moreover, a brief caregiver interview provides an assessment of behaviour changes (Behavioural Disinhibition, Apathy/Inertia, Loss of Sympathy/Empathy, Perseverative/Stereotype, Change in Eating Behaviour; from 0 to 10) and psychotic symptoms (from 0 to 3) usually associated with ALS [37].

In addition, patients underwent a battery of neuropsychological tests to assess specific cognitive domains: executive, attentive, memory, and visuospatial functions.

Executive functions were assessed by means of Wisconsin-Card-Sorting-Test (WCST) [38], the Stroop test [39], and the phonemic and semantic fluency tests [40]. Verbal short-term memory was evaluated by means of Digit Span forward test [41], and long-term verbal and visuo-spatial memory by means of: Rey Auditory Verbal Learning Test (RAVLT) [40] and Rey-Osterrieth Complex Figure Test Different Recall [42].

Non-verbal intelligence was assessed by means of the Raven’s coloured progressive matrices [40], and visuo-spatial functions by means of Clock Drawing Test [26] and Rey-Osterrieth Complex Figure Test Copy [42].

Clinically relevant depression and anxiety symptoms were evaluated through the Hamilton Depression Scale (HDS) [43] and Beck Anxiety Inventory (BAI) [44]. The raw data of the neuropsychological tests were adjusted for age and years of education, according to Italian normative data [40, 45]. Adjusted scores were considered below the cut-off threshold (indicating deficit in cognitive performance) when they were below the fifth percentile from the Italian reference population’s mean.

The cognitive status of the patients was classified according to diagnostic criteria published by Strong et al. [46] into the following categories: (i) ALS with normal cognition (ALS-nci); (ii) ALS with behavioural impairment (ALS-bi); (iii) ALS with cognitive impairment (ALS-ci); (iv) ALS with cognitive and behavioural impairment (ALS-cbi). As said above, patients that fulfilled the criteria for ALS with FTD (ALS–FTD) were not included in the study.

### Assessment of interoceptive sensitivity

The IS was measured by the heartbeat perception task, a validated and reliable task (Cronbach’s alpha = 0.69–0.90) assessing interoceptive sensitivity [47, 48]. In the heartbeat perception task, the participants are required to count, in their mind or whispering, how many beats of their heart they perceive in a specific time frame (2 × 35 s, 2 × 25 s, and 2 × 45 s; trials were randomized across subjects), while being at rest in a comfortable sitting position. The number of beats provided by each participant was compared with the number of beats recorded by an ECG trace (Natus Dantec, Keypoint G4, Planegg, Germany) during the same time interval [47, 48]. Before starting the real task, participants performed a 12-s practice trial. The participants were not aware of the duration of the interval that was going to be presented, or of their accuracy; moreover, during task execution, they could not use strategies, such as taking the beat from their own wrist or chest. Accuracy of heartbeat perception was calculated as the mean score of three heartbeat perception intervals according to the following formula: 1/6 Σ [(1 − (|recorded heartbeats − counted heartbeats|)/recorded heartbeats))] [48]. Using this transformation, the IS score can vary between 0 and 1, with higher scores indicating smaller differences between recorded and perceived heartbeats (i.e., higher accuracy corresponds to higher interoceptive sensitivity).

### Heart rate variability

To account for the possible contribution of autonomic afference to IS, participants’ vagally mediated heart rate variability (HRV) was determined during the second part of the ECG recording session. Specifically, we asked the participants to lay still and to stay awake during a 300-s lasting time interval. The recordings were detrended (smooth priors: *λ* = 500), visually inspected and artefact corrected (adaptive filtering: cubic spline interpolation) before they were subjected to a time-domain analysis. The time-domain analysis was used for the determination of two vagally mediated HRV indices: the root mean square of successive differences between consecutive heartbeats (RMSSD) and the standard deviation of RR intervals (SDRR) [49].

### Evaluation of interoception awareness, alexithymia, and apathy

To assess the interoceptive awareness, all participants underwent the Self Awareness Questionnaire (SAQ) [50], a self-report questionnaire evaluating awareness related to visceral sensations (F1) and to somatosensory sensations (F2). It includes 35 items rated on a 5-point Likert scale (0 = never; 1 = sometimes; 2 = often; 3 = very often; 4 = always); score ranges from 0 to 140, with higher score indicating greater subjective awareness of internal states of own body.

We also employed the Italian version of Multidimensional Assessment of Interoceptive Awareness (MAIA), to assess multiple dimensions of interoception [51, 52]. The MAIA is a self-report questionnaire including 32 items on a 6-points Likert scale, in which the participant must rate “how often each statement applies to you generally in daily life,” with ordinal responses coded from 0 (“never”) to 5 (“always”). This multidimensional tool embeds eight scales: (1) noticing, the awareness of one’s body sensations; (2) not-distracting, the tendency not to ignore or distract oneself from sensations of pain or discomfort; (3) not-worrying, the tendency not to experience emotional distress or worry with sensations of pain or discomfort; (4) attention regulation, the ability to sustain and control attention to body sensation; (5) emotional awareness, the awareness of the connection between body sensations and emotional states; (6) self-regulation, the ability to regulate psychological distress by attention to body sensations; (7) body listening, the tendency to actively listen to the body for insight; and (8) trusting, the experience of one’s body as safe and trustworthy.

To assess the ability to identify and describe emotions, we used the Italian version of the Toronto Alexithymia Scale-20 items (TAS-20) [53], the most widely used self-report tool to assess the Alexithymia construct. The 20 items explore three factors reflecting the main aspects of the alexithymia: difficulty in identifying feelings; difficulty in describing feelings; externally oriented thinking. Each item must be rated on a 5-point Likert scale (from 1 = “completely agree” to 5 = “strongly disagree”). The total score ranges 20–100, with higher scores indicating higher levels of alexithymia. The Italian version of TAS-20 has been demonstrated to show good test–retest reliability (0.86) and adequate internal consistency (Cronbach’s alpha: 0.75) in a wide sample of healthy adults and of medical and psychiatric outpatients (Bressi et al., 1996).

After completing the above tests and scales, all participants fulfilled the Italian version of the self-report version of Apathy Evaluation Scale (AES-S) [54, 55], a questionnaire including 18 items concerning behavioural (items 2, 6, 10, 11, 12), cognitive (items 1, 3, 4, 5, 7, 9, 13, 16), emotional (items 8, 14), and other (items 15, 17, 18) aspects of apathy. All items are scored on 4-point Likert scale (to mean “not at all true,” “slightly true”), “somewhat true” or “very true.” The total score ranges from 18 to 72 points, and higher scores indicate more severe apathy. Patients achieving an AES score ≤ 37 are classified as ALS-apathetic; patients with AES score > 37 as ALS-no-apathetic.

### Statistical analysis

Continuous data were expressed as mean ± standard deviation (SD). Categorical variables were summarized as relative frequencies. We compared the neuropsychological scores achieved by each participant with normative values, to assess prevalence and clinical relevance of cognitive impairment. We compared IS scores of patients and caregivers and IS scores of patients with apathy (ALS-apathetic) or without apathy (ALS-no-apathetic), by Mann–Whitney *U* test, with alpha level set at *p* = 0.05. As a last step, we compared IS as a function of cognitive status defined based on Strong et al. (2017) criteria by means of Kruskal–Wallis test, with alpha level set at *p* = 0.05.

Spearman’s correlations were computed to examine the possible relationship between IS and clinical, psychological, and cognitive variables. The clinical variables were disease duration, disease severity (ALSFRS-R score), disease progression rate [(48—ALSFRS-R score at clinical examination)/disease duration (months)] [56], HRV measures (i.e., RMSSD and SDRR), functional independence (Activities of daily living—ADL, Instrumental activities of daily living—IADL scores); the psychological variables were depression (Hamilton), anxiety (BAI apathy (AES), interoceptive awareness (MAIA), and alexithymia (TAS-20); cognitive variables were ECAS scores, cognitive global functioning (MMSE). Spearman’s correlations were conducted on the ALS patients’ group only. Significance level was set at *p* < 0.05. All analyses were performed using Statistical Package for Social Sciences (SPSS) version 24 (IBM Corp., Armonk, NY, USA). Graphs were elaborated by means of GraphPad Prism version 8.4.2 for Windows (GraphPad Software, La Jolla California USA).

## Results

Fifty-five ALS patients (36 males, mean age 60.3 ± 12.5 years) and 41 caregivers (CG) (17 males, mean age 56.5 ± 10.7) were included in the study. Genetic analysis was negative in all patients but two harbouring the hexanucleotide repeat expansion of the *C9orf72* gene. Routine MRI imaging did not disclose any significant abnormality in ALS patients.

Demographic and clinical data of the study populations are shown in Table 1. Table 2 and Table S1 summarize the results of the cognitive tests.

**Table 1:** Demographic and clinical characteristics of ALS patients

Sample size (*n*)	55
Age at onset	60.3 (12.5)
Gender (F/M)	19/36
Education (years)	10.6 (4.7)
Disease duration (in months)	20.9 (18.8)
Onset region (spinal/bulbar)	47/8
Disease phenotype	
Classic	20/55
Bulbar	6/55
Flail-arm	8/55
Flail-leg	9/55
Pyramidal	7/55
Pure lower motor neuron	4/55
Respiratory	1/55
ALSFRS-R	34.5 (6.8)
ALSFRS-R-rate of progression	0.89 (0.73)
FVC (%)	79.25 (25.1)
ADL	4.7 (1.6)
IADL	5.3 (1.9)

Continuous data are presented as mean (standard deviation) unless otherwise indicated

*Abbreviations*: *ALSFRS-R* Amyotrophic Lateral Sclerosis Functional Rating Scale-Revised, *FVC* forced vital capacity, *ADL* activities of daily life, *IADL* instrumental activities of daily life

**Table 2:** Comparisons of clinical of neuropsychological measures and questionnaires between patients with amyotrophic lateral sclerosis (ALS) and caregivers (CG)

	Total	ALS	CG	*P*-value
Sample size (*n*)	96	55	41	-
Females/males	42/54	19/36	23/18	-
Age (years)	58.9 (10.9)	60.1 (11.1)	57 (10.4)	0.117
Education (years)	11.4 (5.1)	10.4 (4.5)	12.7 (5.5)	0.064
ECAS Total	92.6 (21.8)	88.3 (24.2)	99.5 (15.1)	0.027
ECAS Total ALS	71.2 (15.8)	68.2 (17.5)	75.3 (12.3)	0.098
ECAS Total No ALS	21.6 (5.9)	20.3 (6.4)	23.8 (4.5)	0.003
ECAS language	22.2 (5.3)	21.7 (5.9)	23.1 (4.1)	0.547
ECAS fluency	16.7 (5.6)	16.3 (6.2)	17.4 (4.4)	0.658
ECAS executive functions	31.9 (9.1)	29.9 (9.7)	35.2 (6.8)	0.023
ECAS memory	11.1 (4.8)	9.8 (4.8)	12.9 (4.3)	0.001*
ECAS visuospatial	10.7 (1.8)	10.6 (1.9)	10.8 (1.4)	0.713
MMSE	28.1 (1.9)	27.8 (2.1)	28.5 (1.6)	0.113
ADL	5.1 (1.3)	4.7 (1.6)	5.6 (0.5)	0.026
IADL	5.9 (1.3)	5.3 (1.9)	6.7 (1.9)	0.001*
HDS	6.4 (4.8)	6.9 (4.7)	5.8 (5.1)	0.176
BAI	8.9 (7.8)	9.4 (8.1)	8.3 (7.5)	0.442
AES	28.9 (8.3)	30.2 (9.3)	27 (6.2)	0.114
TAS-20	45.7 (13.3)	46.2 (12.9)	44.9 (14.1)	0.470
MAIA	23.3 (6.2)	24.1 (5.9)	22.6 (6.5)	0.414
SAQ	17.3 (10.8)	17.6 (10.8)	16.8 (10.9)	0.708

Continuous data are presented as mean and standard deviation unless otherwise indicated

*Abbreviations*: *ECAS* Edinburgh Cognitive and Behavioural ALS Screen, *MMSE* Mini Mental Examination State, *ADL* activities of daily life, *IADL* instrumental activities of daily life, *BAI* Beck Anxiety Inventory, *HDS* Hamilton Depression Scale, *AES* Apathy Evaluation Scale, *TAS-20* Toronto Alexithymia Scale, *MAIA* Multidimensional Assessment of Interoceptive Awareness, *SAQ* Self Awareness Questionnaire

^*^Significant differences according to Bonferroni correction (*p* = 0.002; number of comparisons = 19)

**Fig. 1:**
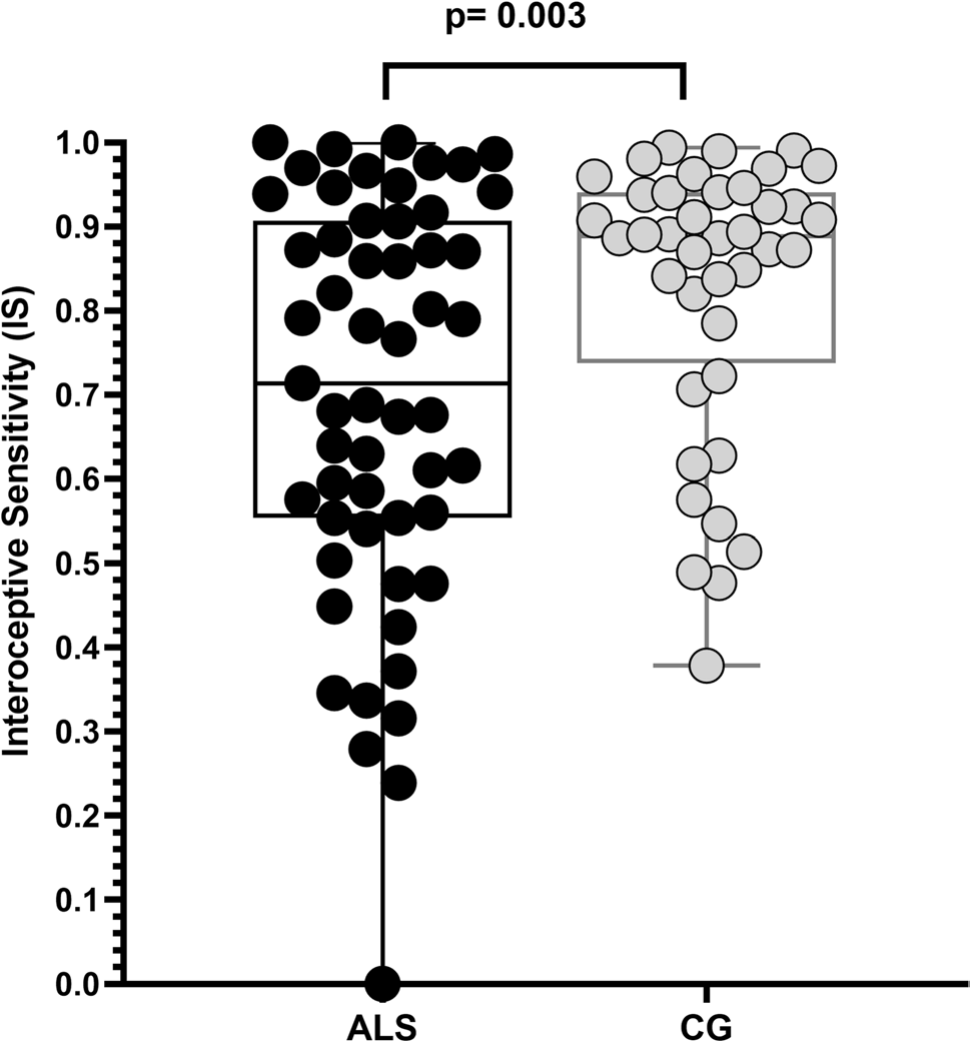
Differences in Interoceptive Sensitivity (IS) during heartbeat count task between ALS patients and caregivers (CG)

ALS patients obtained significantly lower scores on IS than CG (0.68 ± 0.24 vs 0.82 ± 0.16, Mann–Whitney *U* = 746.00, *p* = 0.003) (Fig. 1). No statistically significant differences were found in self-reported measures of anxiety (BAI), depression (Hamilton), apathy (AES), Alexithymia (TAS-20), and interoceptive awareness (MAIA) between two groups (all *p* > 0.05; Table S1). No differences on IS scores were found between ALS-apathetic and ALS-no-apathetic groups.

Moreover, comparisons of IS scores performed by means of non-parametric Kruskal–Wallis test for multiple independent groups as function of Strong classification did not show significant differences among categories (see Table S2).

**Fig. 2:**
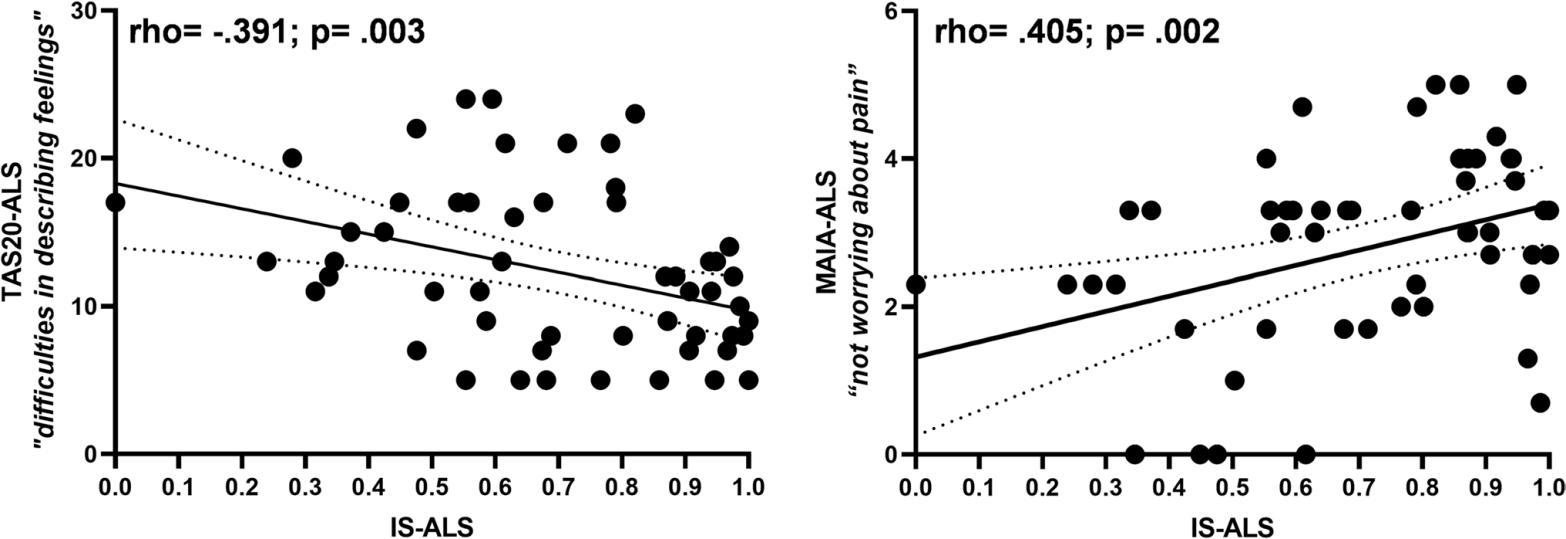
Correlations between interoceptive sensitivity (IS) during heartbeat count task with alexithymia (subscale of Toronto Alexithymia Scale, TAS-20, “difficulties in describing feelings”), left panel, and interoceptive awareness (subscale of Multidimensional Assessment of Interoceptive Awareness, MAIA, “not worrying about pain”), right panel

Lastly, Spearman’s correlation in ALS group (Fig. 2) showed a significant negative association between IS and Alexithymia (score on the *difficulty in describing of feelings subscale* of TAS-20; rho =  − 0.391, *p* = 0.003), a positive association with interoceptive awareness (score on *tendency of worrying about pain subscale* of MAIA; rho = 0.405, *p* = 0.002). No correlations were found between IS and measures of disease severity (ALSFRS-R) and disease progression rate (ALSFRS-R rate). Moreover, no correlations were found between IS and scores of anxiety (BAI), depression (Hamilton), apathy (AES), cognitive tests, and of subscores of ECAS (all *p* > 0.05). Lastly, no significant correlation was observed between IS and HRV measures (all *p* > 0.05). Significant correlations are shown in Fig. 2.

## Discussion

This study represents the first attempt to investigate the interoceptive sensitivity, measured by means of the heartbeat perception task, in patients with ALS. Our findings showed that patients were less accurate on IS task compared to caregivers; altered IS was not associated with disease severity or disease progression rate. Moreover, we found no correlation between IS and psychological self-report measure of anxiety and depression. Instead, we found some correlations with self-report measures of alexithymia and interoceptive awareness. Specifically, in our sample of patients with ALS, low IS was associated with a low ability in describing feelings and a higher tendency to not experience emotional distress or worry with sensations of pain or discomfort. This result is of particular interest because it could reflect a general reduction of interoception mediated by the insula.

This process represents the body-to-brain axis of sensation concerning the state of the internal body and the organs [20]. The central representation and perception of changes in bodily physiology are the basis for emotional feeling states [57]. In this process, the insula is thought to play a key role. Indeed, the connections and activation profile of the insula suggest that it integrates visceral and somatic input and forms a representation of the state of the body [22].

The deficit of IS found in patients with ALS might be an epiphenomenon of a degenerative process involving the insula and a specific behavioural symptom. Indeed, we did not find any correlation between the degree of deficit in IS and the severity of affective symptoms such as apathy, and other psychological symptoms. Similar findings have been described in Parkinson disease, where poor IS on the heartbeat count task did not correlate with non-motor symptoms such as depression, anxiety, apathy, and cognitive functions [58]. Ricciardi et al. [58] suggested that the lack of correlation between self-reported measures of affective status and bodily awareness might reflect separate functional roles for the anterior and posterior insula, being the former involved in cognitive/affective functions and the latter in viscero-sensory/somatosensory awareness [58, 59]. In this perspective, our results might be ascribed to a dissociation between an impairment of IS, as measured by the heartbeat perception task, and a relative sparing of interoceptive processing, more closely related to affective/emotional function, in ALS.

However, our findings also showed the significant association of IS with some dimensions of alexithymia and interoceptive awareness explored by self-report questionnaires. Thus, from a different perspective, this result would demonstrate the significant functional interaction between the two components of interoception mediated by insula (cognitive/affective and viscero-sensory/somatosensory) [20]. In addition, the lack of any correlation between HRV parameters and IS suggested that the impairment of interoception does not seem to be underpinned by an alteration of the afferent autonomic pathway but rather to a degeneration of the central brain networks.

Lastly, we demonstrated that IS is impaired in ALS, regardless of cognitive impairment, or general attentional disturbances, pointing out that behavioural interoceptive alteration is a specific signature of emotion processing deficit. This is in line with other emotion processing disturbances described in ALS, such as recognition of facial expressions of anger, sadness, and disgust that are impaired even when the cognition is preserved [60].

This study has some limitations. First, the relatively small sample size reduced the possibility to explore the association between ALS phenotypes and IS, especially when data were stratified in sub-categories according to the Strong criteria. Second, the lack of longitudinal observations did not allow to verify the reliability of the differences in IS measures between ALS and CG. Lastly, we did not assess variables potentially influencing IS such as personality traits and emotional status.

## Conclusions

In conclusion, our data demonstrated a reduction of interoceptive sensitivity in patients with ALS. This deficit was related neither to presence and severity of cognitive and behavioural symptoms nor to motor disability. Our findings suggested that altered interoceptive accuracy may represent a specific behavioural sign within the spectrum of emotion processing deficits described in ALS. We speculated that impaired interoceptive processing might be linked to insular degeneration rather than to altered autonomic afferences, but this issue remains to be investigated by neuroimaging studies.

## Supplementary Information

Below is the link to the electronic supplementary material.

Supplementary file1 (DOCX 673 KB)

Supplementary file2 (DOCX 18 KB)
