# Supplementary material for: Alteration of interoceptive sensitivity: expanding the spectrum of behavioural disorders in amyotrophic lateral sclerosis
Source: Neurol Sci. 2022 Jun 25;43(9):5403–10. doi: 10.1007/s10072-022-06231-4 (PMC9385786; doi:10.1007/s10072-022-06231-4)
Supplement: Supplementary file 1 — Supplementary file1 (DOCX 673 KB) [file 10072_2022_6231_MOESM1_ESM.docx]

**Table S1.** Frequency of pathological scores (Equivalent scores= 0) on neuropsychological tests and questionnaires.

|  | **Number of Pathological Scores (%)** | |
| --- | --- | --- |
|  | **ALS** | **CG** |
| **ECAS Total** | 14 (25.4) | 0 |
| **ECAS Total ALS** | 10 (18.2) | 0 |
| **ECAS Total No ALS** | 4 (7.3) | 0 |
| **ECAS language** | 10 (14.5) | 0 |
| **ECAS fluency** | 5 (9.1) | 0 |
| **ECAS executive functions** | 6 (10.9) | 0 |
| **ECAS memory** | 3 (5.4) | 0 |
| **ECAS visuospatial** | 6 (10.9) | 0 |
| **Digit Span Forward test** | 0 | - |
| **RAVLT - immediate recall** | 8 (14.5) | - |
| **RAVLT - delayed recall** | 5 (9.1) | - |
| **WCST total score** | 25 (45.4) | - |
| **ROCF - copy** | 14 (25.4) | - |
| **ROCF – delayed recall** | 10 (14.5) | - |
| **FAB** | 15 (27.3) | - |
| **CDT** | 7 (12.7) | - |
| **Verbal fluency (FAS)** | 2 (3.6) | - |
| **Verbal fluency (Semantic)** | 1 (1.8) | - |
| **Stroop – interference** | 0 | - |
| **RCPM** | 5 (9.1) | - |
| **Depression (HDS score >8)** | 34 (61.8) | 10 (24.4) |
| **Anxiety (BAI score > 8)** | 27 (49.1) | 16 (39) |
| **ECAS Behavioural sub-scale (apathy/disinhibition)** | 24 (33.8)/5 (7) | 0/0 |

Abbreviations: RAVLT (Rey Auditory Verbal Learning Test); WCST total score (Wisconsin-Card-Sorting-Test); ROCF (Rey-Osterrieth Complex Figure Test); FAB (Frontal Assessment Battery); RCPM (Raven’s coloured progressive matrices); CDT (Clock Drawing Test); HDS (Hamilton Depression Scale); BAI (Beck Anxiety Inventory).
