# Supplementary material for: Alteration of interoceptive sensitivity: expanding the spectrum of behavioural disorders in amyotrophic lateral sclerosis
Source: Neurol Sci. 2022 Jun 25;43(9):5403–10. doi: 10.1007/s10072-022-06231-4 (PMC9385786; doi:10.1007/s10072-022-06231-4)
Supplement: Supplementary file 2 — Supplementary file2 (DOCX 18 KB) [file 10072_2022_6231_MOESM2_ESM.docx]

|  | **ALS-nci** | **ALS-ci** | **ALS-bi** | **ALS-cbi** | **P** |
| --- | --- | --- | --- | --- | --- |
| **Sample size (n)** | 22 | 8 | 17 | 8 | - |
| **IS (Heart Beat Count score)** | 0.66 (0.30) | 0.70 (0.20) | 0.77 (0.18) | 0.53 (0.33) | .234 |

**Table S2.** Comparisons of Interoceptive Sensitivity as function of Strong classification criteria.

Abbreviations: ALS-nci (patients without cognitive impairment and behavioural disorders); ALS-ci (patients with cognitive impairment); ALS-bi (patients with behavioural disorders); ALS-cbi (patients with both cognitive impairment and behavioural disorders).

Data are expressed as means ± standard deviation and were compared by means of non-parametric Kruskal-Wallis test.
